# Common features of microRNA target prediction tools

**DOI:** 10.3389/fgene.2014.00023

**Published:** 2014-02-18

**Authors:** Sarah M. Peterson, Jeffrey A. Thompson, Melanie L. Ufkin, Pradeep Sathyanarayana, Lucy Liaw, Clare Bates Congdon

**Affiliations:** ^1^Center for Molecular Medicine, Maine Medical Center Research InstituteScarborough, ME, USA; ^2^Graduate School of Biomedical Sciences and Engineering, University of MaineOrono, ME, USA; ^3^Department of Computer Science, University of Southern MainePortland, ME, USA

**Keywords:** microRNA, target prediction, seed match, conservation, free energy, site accessibility, machine learning, computational approaches

## Abstract

The human genome encodes for over 1800 microRNAs (miRNAs), which are short non-coding RNA molecules that function to regulate gene expression post-transcriptionally. Due to the potential for one miRNA to target multiple gene transcripts, miRNAs are recognized as a major mechanism to regulate gene expression and mRNA translation. Computational prediction of miRNA targets is a critical initial step in identifying miRNA:mRNA target interactions for experimental validation. The available tools for miRNA target prediction encompass a range of different computational approaches, from the modeling of physical interactions to the incorporation of machine learning. This review provides an overview of the major computational approaches to miRNA target prediction. Our discussion highlights three tools for their ease of use, reliance on relatively updated versions of miRBase, and range of capabilities, and these are DIANA-microT-CDS, miRanda-mirSVR, and TargetScan. In comparison across all miRNA target prediction tools, four main aspects of the miRNA:mRNA target interaction emerge as common features on which most target prediction is based: seed match, conservation, free energy, and site accessibility. This review explains these features and identifies how they are incorporated into currently available target prediction tools. MiRNA target prediction is a dynamic field with increasing attention on development of new analysis tools. This review attempts to provide a comprehensive assessment of these tools in a manner that is accessible across disciplines. Understanding the basis of these prediction methodologies will aid in user selection of the appropriate tools and interpretation of the tool output.

## Introduction

MicroRNAs (miRNAs) are ~22 nucleotide long endogenous RNA regulators of gene activity at the post-transcriptional level. Since the discovery of miRNAs in 1993 (Lee et al., 1993; Wightman et al., 1993), miRNAs have been identified as key regulators of proliferation, differentiation, and cell death in both normal and aberrant pathways (Friedman and Jones, 2009; Garzon et al., 2009; Ambros, 2011; Starega-Roslan et al., 2011; Iuliano et al., 2013). MiRNAs function by targeting complementary sequences in mRNA transcripts, usually in the 3′ untranslated region (3′ UTR), and prevent protein synthesis by inhibiting translation or inducing target degradation. Identification and validation of miRNA:mRNA target interactions is the foundation for discerning the role of miRNAs in the broader context of miRNA regulatory networks governing biological processes.

An extremely large number of potential target sites exists for any given miRNA, and the process of validating a potential miRNA target in the laboratory is time consuming and costly. A computational approach to prediction of miRNA targets facilitates the process of narrowing down potential target sites for experimental validation. Computational approaches model how miRNAs target specific mRNAs and an increasing collection of tools is available, each with a distinct approach to miRNA target prediction. While it may be advantageous to have access to a range of tools with different capabilities, the user is confronted with an important choice in deciding which tool to use.

Although recent reviews exist on human miRNA target identification tools (Reyes-Herrera and Ficarra, 2012; Dweep et al., 2013; Vlachos and Hatzigeorgiou, 2013), this review attempts to present the computational aspects of these tools at a level that is both accurate and accessible across disciplines. Therefore, this review highlights the common features (see Common Features of miRNA Target Prediction Tools) and less common features (see Less Common Features of miRNA Target Prediction Tools) used in developing miRNA target prediction tools, followed by a review of common tools (see Review of Commonly Used miRNA Target Prediction Tools), a summary of excluded tools (see Brief Summary of Tools Excluded from this Review), and lastly a discussion of all of these tools (see Discussion). We have included special consideration of features such as tool maintenance and user-friendliness. We note here the existence of combinations of one or more of these tools into integrated tools. While an evaluation of integrated tools is outside the scope of this review, knowledge of the strengths and limitations of individual component tools is certainly relevant to the user assessment of an integrated tool. Our goal is to provide information for researchers to make an informed decision about which tool to use based on the needs of a particular project.

## Common features of miRNA target prediction tools

There are four commonly used features for miRNA target prediction tools: seed match, conservation, free energy, and site accessibility. These will be described in the following sections.

### Seed match

**Figure 1:**
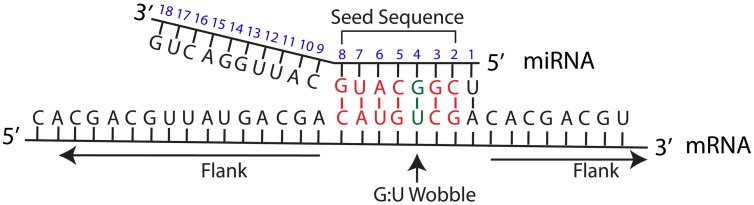
**miRNA:mRNA target interaction**. Schematic overview of a miRNA interaction with its mRNA target. MiRNA position number is shown in blue. The seed sequence refers to nucleotides in miRNA position number 2–8. Flank refers to the mRNA sequence on either side of the region corresponding to the miRNA seed sequence. WC matches in the seed sequence are shown in red, and an example of G-U wobble in the seed sequence is shown in green.

The seed sequence of a miRNA is defined as the first 2–8 nucleotides starting at the 5′ end and counting toward the 3′ end (Lewis et al., 2003) (Figure 1). For most tools, a seed match is a Watson-Crick (WC) match between a miRNA and its target in the seed sequence. A WC match between a miRNA and mRNA nucleotide occurs when adenosine (A) pairs with uracil (U) and guanine (G) pairs with cytosine (C). A perfect seed match between the miRNA and the mRNA target has no gaps in alignment within the WC matching.

There are several types of seed matches that can be considered depending on the algorithm. The following types are the main types of seed matches (Lewis et al., 2003, 2005; Brennecke et al., 2005; Krek et al., 2005):

1. 6mer: A perfect WC match between the miRNA seed and mRNA for six nucleotides.
2. 7mer-m8: A perfect WC match from nucleotides 2–8 of the miRNA seed.
3. 7mer-A1: A perfect WC match from nucleotides 2–7 of the miRNA seed in addition to an A across from the miRNA nucleotide 1.
4. 8mer: A perfect WC match from nucleotides 2–8 of the miRNA seed in addition to an A across from the miRNA nucleotide 1.

### Conservation

Conservation refers to the maintenance of a sequence across species. Conservation analysis may focus on regions in the 3′ UTR, the 5′ UTR, the miRNA, or any combination of the three. In general, there is higher conservation in the miRNA seed region than in the non-seed region (Lewis et al., 2003). In a small proportion of miRNA:mRNA target interactions, there is conserved pairing at the 3′ end of the miRNA which can compensate for seed mismatches, and these sites are called 3′ compensatory sites (Friedman et al., 2009). In the context of predicting miRNA targets in 3′ UTRs, conservation analysis may provide evidence that a predicted miRNA target is functional because it is being selected for. Additionally, there is increasing interest in conservation analysis of the genomic regions flanking the miRNA gene and miRNA target genes. As examples, conservation analysis has been applied to the promoter regions of miRNAs and their target genes (Fujiwara and Yada, 2013), and to the co-localization of independently transcribed miRNAs and flanking protein coding genes (Ohler et al., 2004). Thus, the role of conservation in miRNA target prediction is broad and analysis of conserved elements can be incorporated into miRNA target prediction in a variety of ways.

### Free energy

**Figure 2:**
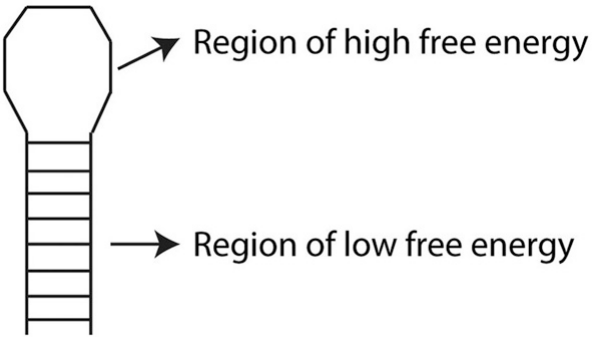
**Schematic overview of free energy (Δ*G*) analysis of predicted RNA hybridization structure**. A hairpin loop is shown with the loop corresponding to a region of high free energy (a positive Δ*G*) and the stem corresponding to a region of low free energy (a negative Δ*G*).

Free energy (or Gibbs free energy) can be used as a measure of the stability of a biological system. If the binding of a miRNA to a candidate target mRNA is predicted to be stable, it is considered more likely to be a true target of the miRNA. Given the difficulty in measuring free energy directly, usually the change in free energy during a reaction is considered (Δ*G*). Since reactions with a negative Δ*G* have less energy available to react in the future, they result in systems with increased stability. By predicting how the miRNA and its candidate target hybridize, regions of high and low free energy can be inferred (Figure 2) and the overall Δ*G* can be used as an indicator of how strongly bound they are (Yue et al., 2009).

### Site accessibility

Site accessibility is a measure of the ease with which a miRNA can locate and hybridize with an mRNA target. Following transcription, mRNA assumes a secondary structure (Mahen et al., 2010) which can interfere with a miRNA's ability to bind to a target site. MiRNA:mRNA hybridization involves a two-step process in which a miRNA binds first to a short accessible region of the mRNA. The mRNA secondary structure then unfolds as the miRNA completes binding to a target (Long et al., 2007). Therefore, to assess the likelihood that an mRNA is the target of a miRNA, the predicted amount of energy required to make a site accessible to a miRNA can be evaluated.

## Less common features of miRNA target prediction tools

The features discussed above are those most commonly incorporated into miRNA target prediction tools. As new advances are made in the characterization of miRNA:mRNA target interactions, additional features are incorporated. These might be used to predict the effectiveness of the target or directly incorporated into the target prediction itself. Target-site abundance is a measure of how many target sites occur in a 3′ UTR (Garcia et al., 2011). Local AU content refers to the concentration of A and U nucleotides flanking the corresponding seed region of the miRNA (Friedman et al., 2009; Betel et al., 2010). GU wobble in the seed match refers to the allowance of a G pairing with a U instead of a C (Doench and Sharp, 2004). 3′ compensatory pairing refers to base pair matching with miRNA nucleotides 12–17. Seed pairing stability is the calculated free energy of the predicted duplex (Garcia et al., 2011). Position contribution analyzes the position of the target site within the mRNA (Grimson et al., 2007). Machine-learning approaches use training data to develop a model of miRNA targets, and then use the model as part of the miRNA-prediction process. Machine-learning techniques are likely to use more features in their predictions because they can be trained to determine the predictive power of each feature on positive and negative datasets. A machine-learning approach used by several of these tools is support vector machines (SVM). Tools that use SVM are noted.

## Review of commonly used miRNA target prediction tools

In this section, we outline 10 popular miRNA target prediction tools, using the characteristics previously described. A summary table comparing these tools is provided in the Comparison of miRNA Target Prediction Tools section (Table 11).

### miRanda

miRanda (Enright et al., 2003) is one of the earlier miRNA target predictors, but it has continued to be updated (Table 1). Although it was originally used to find targets in *Drosophila*, the algorithm is not limited in this regard and was subsequently used to predict targets in humans (John et al., 2004). Although miRanda is available online as part of the miRanda-mirSVR tool (reviewed below), to be used on its own it must be downloaded.

**Table 1:** **Profile of miRanda**.

Website	http://www.microrna.org/ (source code)
Version	Current version is 3.3a, last updated 8/2010
Input	User-supplied miRNA sequence and UTR sequence for command line
Organisms	Any
User adjustability	Free energy threshold, alignment threshold, weight of seed region, and gap penalty
Features	Seed match, conservation, and free energy

miRanda uses a three-step analysis. First, the miRNA sequences provided as input are scanned against user-provided 3′ UTRs to check for WC matches. The free energy of each miRNA:mRNA target pair that exceeds a threshold matching score is calculated. Each target that has a predicted free energy below a threshold is then passed to the last step. Finally, conservation is used as a final filter. However, miRanda considers conservation of both binding site and position. The remaining candidates are scored based on how well they match the miRNA. A predicted target can be ranked high in the results by either obtaining a high individual score or by having multiple predicted sites. Unlike most miRNA target predictors, miRanda considers matching along the entire miRNA sequence (Enright et al., 2003). It takes the seed region into account by weighting matches in the seed region more heavily. Matches are allowed to contain limited G-U wobble pairs and insertions or deletions (indels). Free energy is calculated by predicting the folding of the miRNA:mRNA hybrid using the Vienna package (Hofacker et al., 1994). Although this is a common method, it ignores any additional protein interaction, such as with the RNA-induced silencing complex (Enright et al., 2003).

miRanda was written in C and provided as source code. It is relatively easy to compile and run. Nevertheless, both this step and the requirement to run miRanda using the command line will present a technical barrier for many users. However, for more advanced users, miRanda provides a number of adjustable parameters that may be helpful in investigating particular miRNA targets.

### miRanda-mirSVR

miRanda-mirSVR (Betel et al., 2010) is an online tool that combines two approaches (Table 2). miRanda is used to identify candidate target sites and mirSVR is used to score them. The results are pre-computed, with no option to supply new data. Identification of candidate target sites is described in the section on miRanda. However, scoring is performed using mirSVR, a support vector regression (SVR) approach that is similar to SVM. However, an SVR uses real valued outputs rather than classifying candidates into discrete groups. These are used by mirSVR to compute a score that represents the effect a miRNA may have on expression. mirSVR was trained on nine miRNA transfection experiments performed on HeLa cells (Betel et al., 2010) and incorporates a number of other features that it found relevant: site accessibility, AU flanking content, position of the target site within the 3′ UTR, and UTR length.

**Table 2:** **Profile of miRanda-mirSVR**.

Website	http://www.microrna.org/
Version	Current version is 3.3a, last updated 8/2010, uses miRBase version 15
Input	miRNA identifier or gene name
Organisms	Humans, rats, mice, flies, and worms
User adjustability	None
Features	Seed match, conservation, free energy, site accessibility, and others

Although miRanda-mirSVR possesses many of the same capabilities as other prediction tools, the mirSVR score is particularly useful in that it provides an indication of the strength of a miRNA's regulatory effect. Unfortunately, the site is maintained erratically, and does not always use the latest version of miRBase (Kozomara and Griffiths-Jones, 2011) for its predictions. This is a particular problem in terms of using the latest nomenclature when searching for a particular miRNA. Nevertheless, the mirSVR score is a unique and useful capability, and the website is easy to navigate. In addition, the site provides analysis of miRNA expression by tissue and links to miRBase and miRo (The miR-Ontology Database) (Lagana et al., 2009) for more information about a miRNA of interest.

### TargetScan

TargetScan (Lewis et al., 2005; Grimson et al., 2007; Friedman et al., 2009; Garcia et al., 2011) allows the user to search by miRNA name, gene name, or from broadly conserved, conserved, or poorly conserved miRNA families across several species (Table 3). The output screen ranks predicted targets by either the predicted efficacy of targeting (context+ scores) or the probability of conserved targeting (P_CT_). For conservation, the conservation of a 3′ UTR is first determined followed by analysis of a specific k-mer (8mer, 7mer-m8, or 7mer-1A). Since one 3′ UTR can contain multiple target sites, an aggregate P_CT_ is provided. For each type of k-mer, the number is provided for that target and whether or not it is considered a conserved site or a poorly conserved site. Furthermore, there is a link to the 3′ UTR of the gene that demonstrates the conserved seed sequence (Friedman et al., 2009). The context+ score demonstrates the probability of a given target as being effectively targeted. Scoring for this feature was derived from experimental results. Several features are included when defining the score, such as 3′ compensatory pairing, local AU content, and position contribution (Grimson et al., 2007; Garcia et al., 2011).

**Table 3:** **Profile of TargetScan**.

Website	http://www.targetscan.org
Version	Current version is 6.2, last updated 6/2012
Input	miRNA name, gene name or miRNA family
Organism	Mammals, flies, and worms
User adjustability	No
Features	Seed match and conservation

TargetScan is easy to use and actively maintained. It does not require the input of sequences or the adjustment of advanced settings, which could potentially be viewed as an advantage for novice users or a drawback for advanced users.

### DIANA-microT-CDS

DIANA-microT-CDS (Maragkakis et al., 2009; Reczko et al., 2012; Paraskevopoulou et al., 2013) is the latest version of DIANA-microT, which was one of the first miRNA target prediction systems to predict targets in humans (Table 4). The new version incorporates a machine-learning approach to identify the most relevant features extracted from photoactivatable-ribonucleoside-enhanced crosslinking and immunoprecipitation (PAR-CLIP) data. These data provide DIANA-microT-CDS the opportunity to learn the features associated with miRNA whose binding location is directly known in both coding sequences (CDS) and 3′ UTR. Additionally, microarray expression data were used to learn the contribution of multiple sites in a target (Reczko et al., 2012). For both regions, the most important features were the binding category weight (as an estimate of the efficiency of binding based primarily on matching in an extended seed sequence), distance to the nearest end of the region (CDS or 3′ UTR), distance to an adjacent binding site, the predicted free energy of the hybrid [using RNAhybrid, reviewed in the RNAhybrid section (Rehmsmeier et al., 2004; Kruger and Rehmsmeier, 2006)], conservation, and AU content. Additionally, the accessibility of the 3′ UTR was found to be relevant, which was predicted using Sfold (Chan et al., 2005). DIANA-microT-CDS uses individual models for miRNA binding in both the CDS and the 3′ UTR to separately score targeting in each region before combining both into a single score. Therefore, DIANA-microT-CDS is able to predict targeting in cases even when there is no site in the 3′ UTR but at the same time can rank predictions higher that have multiple sites in both regions (Reczko et al., 2012).

**Table 4:** **Profile of DIANA-microT-CDS**.

Website	http://www.microrna.gr/microT-CDS
Version	Current version is 5.0, last updated 7/2012, uses miRBase version 18
Input	miRNA name, gene name, Ensembl ID, KEGG description, or some combination of these
Organisms	Humans, mice, flies, and worms
User adjustability	None
Features	Seed match, conservation, free energy, site accessibility and target-site abundance

DIANA-microT-CDS can be searched by miRNA name, gene name, Ensembl ID, KEGG description, or a combination of these (Paraskevopoulou et al., 2013). Species are specified as part of the miRNA or Ensembl ID, which is somewhat awkward considering that it does not make clear what species are available. However, if it is not specified, DIANA-microT-CDS will ask for clarification. Even with this slight issue, DIANA-microT-CDS is one of the easiest tools to use. The results include the predicted target location, binding type, score, conservation, and links to Ensembl, miRBase, and PubMed that relate to the search (Paraskevopoulou et al., 2013). Additionally, it shows when the target was also predicted by miRanda or TargetScan or was experimentally verified in TarBase (Paraskevopoulou et al., 2013). For advanced users, a Taverna plugin allows more options and a non-web interface.

### MirTarget2

MirTarget2 (Wang, 2008; Wang and El Naqa, 2008) makes miRNA target predictions using SVM and features extracted from a large microarray training dataset (Linsley et al., 2007) (Table 5). This machine-learning approach confirmed the use of several popular prediction features and identified new features significantly correlated with miRNA:mRNA target interactions. The training features used include seed conservation, seed match specifically in positions 2–8, base composition in the regions flanking the seed pairing sites, secondary structure (incorporating site accessibility and free energy), and location of the site within the 3′ UTR. MirTarget2 was created in conjunction with miRDB (Wang, 2008), and MirTarget2 predictions are available in miRDB. miRDB is a Wikipedia-like functional annotation database for mature miRNA with integration of high throughput automated annotations and manual annotations from individual researchers (Wang, 2008). Seed conservation is incorporated and scored by comparing human, mouse, rat, dog, and chicken orthologs, but is not required. One potential limitation of this program is that the training dataset included only 3′ UTR sequences with a single seed pairing site, as opposed to multiple target sites. The rationale for this was to minimize complications from determining the contribution of each binding site. While this is an understandable choice, it also presents a theoretical limitation of the training dataset given that target-site abundance can alter the likelihood of miRNA:mRNA interactions (Garcia et al., 2011).

**Table 5:** **Profile of MirTarget2**.

Website	Predicted targets imported into miRDB at http://mirdb.org
Version	No version number available, last updated 4/2012
Input	miRNA name, gene name, National Center for Biotechnology Information (NCBI) RefSeq mRNA accession number, gene ID or GenBank accession number
Organisms	Humans, mice, rats, dogs, and chickens
User adjustability	Adjustable and default screening options are available for the target mining option
Features	Seed match, conservation, free energy, site accessibility and others (SVM based)

Overall, miRDB is actively maintained and user friendly. Predicted miRNA:mRNA target interactions can be searched by miRNA or by mRNA. There is also a target mining option with adjustable and default screening options. This is useful because large numbers of targets are predicted for some miRNAs (e.g., 280 targets for miR-143-3p and 542 targets for miR-145-5p) (Wang, 2008). There are also links to precompiled pathways for miRNA regulators from PANTHER (Protein ANalysis THrough Evolutionary Relationships) (Mi et al., 2013).

### rna22-GUI

rna22-GUI (Loher and Rigoutsos, 2012) is based on rna22 (Hofacker et al., 1994; Miranda et al., 2006), an older target prediction tool (Table 6). rna22 used pattern discovery to identify target islands and evaluate the free energy of paired target islands and candidate miRNAs. Unlike rna22, which required a user-provided miRNA and target sequence, rna22-GUI offers the ability to search by miRNA, gene ID, transcript ID, or gene name. Search results can be presented as a cDNA map, which shows the predicted nucleotide interactions of each of the miRNA:mRNA target interaction sites or as a table that lists these predicted duplexes.

**Table 6:** **Profile of rna22-GUI**.

Website	https://cm.jefferson.edu/rna22v1.0/
Version	No version number available, developed in 2012, uses miRBase version 16, Ensembl release 62
Input	miRNA name, Ensembl gene ID, Ensembl transcript ID, or gene name
Organism	Humans, mice, flies, and worms
User adjustability	None
Features	Seed match and free energy

While some users may gravitate toward the graphical representation of miRNA:mRNA target interactions, novice users may find the map complicated and difficult to navigate.

### TargetMiner

TargetMiner (Bandyopadhyay and Mitra, 2009) is an SVM-based classifier for identifying potential seed sites between a user-provided miRNA and mRNA of choice (Table 7). The user can search as many miRNAs and targets as desired when uploading the input file. The user is provided with the type of seed match, position, and how many of those sites are found within the sequence. The tool is based on machine learning from negative and positive training data in order to provide more accurate seed match predictions between a miRNA and its target. The positive training data was a set of 289 miRNA transcript pairs extracted from the miRecords database (Xiao et al., 2009). The negative training data was selected from a pooled dataset of pairs of miRNAs and predicted targets by identification of overlapping false positive pairs generated from multiple target prediction algorithms. Tissue specific non-target pairs were then identified by using expression profiling data. While the SVM-based classifier includes consideration of multiple common features inside and outside of the seed region, the output provides the user with information only about the predicted seed match.

**Table 7:** **Profile of TargetMiner**.

Website	http://www.isical.ac.in/~bioinfo_miu/targetminer20.htm
Version	No version number available, developed in 2009, downloadable list of predictions last updated 5/2012
Input	miRNA name and NCBI RefSeq mRNA accession number in a user-provided input file
Organism	Any
User adjustability	No
Features	Seed match, conservation, free energy, site accessibility, target-site abundance and others

Novice users may be dissuaded from using this tool due to the requirement for a preparation of an input file. For advanced users, a downloadable executable version of TargetMiner is available.

### SVMicrO

SVMicrO (Liu et al., 2010) is a machine-learning approach to miRNA target prediction (Table 8). The authors used a relatively large positive training data set spanning multiple species. Since they were not able to find experimentally validated negative data, they used expression data. The authors identified 113 possible features of the miRNA binding site, along with 30 possible features of the 3′ UTR as a whole. They ran a minimal redundancy maximal relevance algorithm with the training data to determine which of these features were the best predictors of miRNA regulation. This left them with 21 site-related and 18 UTR-related features, although these features are more granular than those discussed in the Common Features of miRNA Target Prediction Tools section. For example, 8mer and 7mer seed matches are considered separate features. SVMicrO uses these features to predict candidate miRNA:mRNA target pairs. Five features (seed match, conservation, free energy, site accessibility, and target-site abundance) were found to be important in predicting miRNA targets, but the training data allowed these to be defined with a tighter focus.

**Table 8:** **Profile of SVMicrO**.

Website	http://compgenomics.utsa.edu/svmicro.html
Version	No version number available, developed in 2010
Input	User-supplied sequences
Organisms	Any
User adjustability	None
Features	Seed match, conservation, free energy, site accessibility and target-site abundance

SVMicro's use of numerous granular features in predicting miRNA:mRNA target pairs is powerful. Furthermore, given SVMicrO's relatively large training data set, these features may be useful to other systems. However, usability is currently a limitation. The user needs to build a database containing the UTR to search combined with phastCons conservation scores (Siepel et al., 2005). However, there is no documentation for how this should be done. Furthermore, SVMicrO will only install easily on a 32-bit Linux operating system. It also assumes that the system echo command will be used instead of a built-in shell version. Although it is possible to install it on a 64-bit system, it will be necessary to modify the source. These issues constitute significant obstacles for users unfamiliar with these steps.

### Probability of interaction by target accessibility (PITA)

PITA (Kertesz et al., 2007) uses target-site accessibility as the major feature for miRNA target prediction (Table 9). This is based on the important observation that there is preferential and conserved positioning of target sites in more accessible regions of the UTR. PITA first identifies a potential site by seed match criteria, and then considers site accessibility by computing a free energy score based on the difference between the gain of free energy associated with miRNA:mRNA target duplex formation and the free energy cost of unpairing the target to make it accessible. Next, target-site abundance is considered by combining the site accessibility scores for the same miRNA to identify a total interaction score for the miRNA and UTR. Several options are available for interaction with PITA on the tool website. These include downloading PITA catalogs of predictions and searching predictions by miRNA or by target gene. PITA can also predict which miRNA might target a user-provided UTR sequence. This feature is advantageous for an advanced user who wishes to evaluate the 3′ UTR of a novel gene or the 5′ UTR of a gene of interest. With the web version of PITA, users can choose from a selection of pre-set seed match criteria including minimum seed size, allowance of a single G-U wobble or mismatch, minimum seed conservation, and flank settings. Advanced users, however, have the option of downloading the PITA executable with expanded flexibility and advanced parameter setting (Kertesz et al., 2007).

**Table 9:** **Profile of PITA**.

Website	http://genie.weizmann.ac.il/pubs/mir07/
Version	Current version is 6, last updated in 2008, uses miRBase version 11 and genome versions ce6, dm3, mm9, and hg18
Input	For the web interface, the user provides miRNA and gene names or NCBI RefSeq mRNA accession numbers. A web-based option is available for user-provided sequence data, and a downloadable executable version is available
Organisms	Humans, mice, flies, and worms
User adjustability	Seed size, wobble or mismatch, conservation, and inclusion of a flank region
Features	Seed match, conservation, free energy, site accessibility and target-site abundance

Overall, PITA is a user-friendly tool for both novice and advanced users. While novice users may prefer not to have to enter any seed match parameters, suggested choices for seed parameter settings are provided in the FAQ link. One major limitation of the web version of PITA is that the predictions are based on miRNA sequences from miRBase version 11 (Kertesz et al., 2007). (As of 6/2013, miRBase version 20 has been released, which contains several thousand new entries.) While the reliance on PITA website administrators for continual updates with the latest version of miRBase may be circumvented by downloading the PITA executable, most users are likely to prefer the web-based application.

### RNAhybrid

RNAhybrid (Rehmsmeier et al., 2004; Kruger and Rehmsmeier, 2006) considers the free energy between a miRNA and an mRNA with a user-defined seed region (Table 10). This tool provides a number of advanced settings including specification of hits per target, helix constraints, maximal internal loop size, maximal bulge loop size and maximum free energy cutoff, which are described in detail in the tool manual available at the RNAhybrid website. RNAhybrid can also assign a *p*-value for the miRNA:mRNA interaction based on the number of binding sites within the 3′ UTR sequence, which is a measure of target-site abundance.

**Table 10:** **Profile of RNAhybrid**.

Website	http://bibiserv.techfak.uni-bielefeld.de/rnahybrid/
Version	No version number available, developed in 2004
Input	User-supplied data for miRNA sequence and mRNA sequence
Organism	Any
User adjustability	Requires advanced user for specification of parameters
Features	Seed match, free energy, and target-site abundance

This tool is intended for advanced users because it requires the input of the miRNA sequence and the mRNA 3′ UTR sequence (both in FASTA format) and has options for manipulation of several advanced settings that are specific to this tool.

### Comparison of miRNA target prediction tools

For ease of comparison, a summary table of reviewed tools is provided (Table 11).

Table 11**Summary table of miRNA target prediction tools**.

**Table:** 

**FEATURES USED IN miRNA TARGET PREDICTION**							
**Tool name**	**Seed match**	**Conservation**	**Free energy**	**Site accessibility**	**Target-site abundance**	**Machine learning**	**References**
miRanda	X	X	X				Enright et al., 2003; John et al., 2004
miRanda-mirSVR	X	X	X	X		X	Betel et al., 2010
TargetScan	X	X					Lewis et al., 2005; Grimson et al., 2007; Friedman et al., 2009; Garcia et al., 2011
DIANA-microT-CDS	X	X	X	X	X	X	Maragkakis et al., 2009; Reczko et al., 2012; Paraskevopoulou et al., 2013
MirTarget2	X	X	X	X		X	Wang, 2008; Wang and El Naqa, 2008
RNA22-GUI	X		X				Hofacker et al., 1994; Miranda et al., 2006; Loher and Rigoutsos, 2012
TargetMiner	X	X	X	X	X	X	Bandyopadhyay and Mitra, 2009
SVMicrO	X	X	X	X	X	X	Liu et al., 2010
PITA	X	X	X	X	X		Kertesz et al., 2007
RNAhybrid	X		X		X		Rehmsmeier et al., 2004; Kruger and Rehmsmeier, 2006

**Table:** 

**TOOL AVAILABILITY AND USER FEATURES**						
**Tool name**	**Website**	**Online use**	**Source code**	**User adjustability**	**User-supplied data required**	**User level**
miRanda	http://www.microrna.org/		X	X	Sequences	Advanced
miRanda-mirSVR	http://www.microrna.org/	X				All
TargetScan	http://www.targetscan.org	X				All
DIANA-microT-CDS	http://www.microrna.gr/microT-CDS	X				All
MirTarget2	http://mirdb.org	X		X		All
RNA22-GUI	https://cm.jefferson.edu/rna22v1.0/	X				Intermediate
TargetMiner	http://www.isical.ac.in/~bioinfo_miu/targetminer20.htm	X	X		Input file	Intermediate
SVMicrO	http://compgenomics.utsa.edu/svmicro.html	X	X		Sequences	Expert

PITA	http://genie.weizmann.ac.il/pubs/mir07/	X	X	X		All
RNAhybrid	http://bibiserv.techfak.uni-bielefeld.de/rnahybrid/	X	X	X	Sequences	Advanced

All reviewed tools are freely available for academic use. All tools are actively maintained with updates in the past 5 years, with the exception of PITA and RNAhybrid.

## Brief summary of tools excluded from this review

Space prevents inclusion of an exhaustive listing of miRNA target prediction software, although some of the original miRNA target prediction tools warrant mention, such as Pictar (Krek et al., 2005) (based on data that is over 10 years out of date) and rna22 (Hofacker et al., 1994; Miranda et al., 2006) (not functional). Other tools, such as NBmiRTar (Yousef et al., 2007), were excluded based on the use of data that is over 5 years out of date, without an option for the inclusion of updated data. Tools that are not currently operational, such as miTarget (Kim et al., 2006) and MicroInspector (Rusinov et al., 2005), are also excluded. Some tools, such as Genmir++ (Huang et al., 2007) and HuMiTar (Ruan et al., 2008), were excluded on the basis of requiring additional proprietary software and/or expertise for use, which make them inaccessible to the average user. MicroCosm Targets uses the miRanda algorithm and was not separately reviewed (Griffiths-Jones et al., 2008). Space also prevents the review of miRNA target prediction programs exclusively for species other than humans. Lastly, while the emerging field of integrated tools is outside of the scope of this review, elucidation of the strengths and limitations of the component tools is highly relevant to the overall user assessment of an integrated tool.

## Discussion

Identifying the target of a specific miRNA is one approach for discovering the role of the miRNA in normal or aberrant biological processes. Possibly thousands of targets exist, however, for any single miRNA. Over the last 17 years, several tools have been developed to address this complex issue. Each of these projects has contributed to our understanding of the relationship between miRNA and mRNA targets and how that relationship can be used to make accurate predictions.

A recently published study by one of the authors exemplifies how a miRNA target prediction tool can be used to generate candidate targets for subsequent experimental validation (Favreau et al., 2012). While studying the functional role of miR-199b-5p in acute myeloid leukemia (AML), TargetScan was used to examine potential targets of miR-199b-5p based on seed match and conservation. Two highly conserved targets, Podocalyxin (PODXL) and Discoidin Receptor 1 (DDR1), are listed as predicted targets of miR-199b-5p by TargetScan (Garcia et al., 2011; Favreau et al., 2012). Experimental validation via transfection of miR-199b-5p mimics in cell lines confirmed that PODXL and DDR1 are targets of miR-199b-5p at both the mRNA and protein levels (Favreau et al., 2012). Further validation by 3′ UTR luciferase assays confirmed that PODXL and DDR1 are true targets of miR-199b-5p (Favreau et al., 2012).

Although each of the reviewed tools has predictive power, they all have limitations based on the weighting and incorporation of features into the tool. If solely relying on seed match for target identification, a method would exclude whether or not the sequence is conserved or if the site is accessible and thermodynamically favorable. There is evidence that many non-conserved binding sites in the 3′ UTR are functional (Farh et al., 2005). Therefore, relying solely on conservation-based miRNA target prediction systems would be unlikely to capture these miRNA:mRNA interactions (Farh et al., 2005; Witkos et al., 2011). Free energy calculations rely on empiric measurements that may not be complete or accurate (Mathews et al., 1999; Wuchty et al., 1999). The quality of the data used in the free energy calculation can thus be a source of error. Furthermore, relying on a predicted free energy release does not guarantee that the interaction exists. It is important to consider the limitation of each of these common features and how they are used in the context of each tool.

Even though these tools use a combination of features to compensate for the limitations of each feature alone, each tool has its own strengths and limitations. Currently, three of these projects stand out in terms of their wide range of capabilities, ease of use, relatively current input data, and maintenance of the software. These are DIANA-microT-CDS, miRanda-mirSVR, and TargetScan. All of these projects have received periodic updates over the last several years and are easy to use. DIANA-microT-CDS uses the most current data out of any of the tools reviewed (miRBase version 18). Furthermore, it is able to make predictions into the CDS in addition to the 3′ UTR. Although miRanda-mirSVR uses a somewhat older version of miRBase (miRBase version 15) than DIANA-microT-CDS, its mirSVR score is a unique capability that provides a meaningful indication of the degree of regulation. Most other tools only provide a score of the result's significance (which is also provided by miRanda-mirSVR). In both DIANA-microT-CDS and miRanda-miRSVR, conservation is a feature not a filter, which increases sensitivity to miRNA targets that are lineage specific. TargetScan is based on only slightly older data (miRBase version 17) than DIANA-microT-CDS. Although it applies a conservation filter, it does allow for poorly conserved targets. Similar to miRanda-mirSVR, it also considers the additional feature of A-U content in the regions flanking the seed region.

Among the remaining target prediction tools reviewed, miRanda is still a widely-used tool even though it needs to be downloaded to be used and it lacks the additional mirSVR score available in miRanda-mirSVR, which may be desirable. rna22-GUI offers a graphical representation of miRNA:mRNA target interactions, but it is based on the original rna22 program and therefore does not incorporate recent advancements in the understanding of miRNA:mRNA target interactions. TargetMiner requires a user-supplied input file and the tool output is limited to seed match characterization. RNAhybrid requires an advanced user due to user-supplied input, adjustment of complex settings, and lack of default values for novice users. The web version of PITA is based on data that is over 5 years out of date, but a downloadable version compatible with user-provided data is available as an alternative option. The final two remaining reviewed tools, SVM-based MirTarget2 and SVMicrO, are machine-learning tools which hold the promise of learning the subtle contributions of many individual features and using them to make more accurate predictions. As more of these features are elucidated and as more positive and negative targets are validated, the promise of machine-learning approaches to use these features to accurately predict targets comes closer to fruition. At present, these last two machine-learning tools do not display a clear advantage over the tools reviewed above and are inherently limited by the lack of extensive positive and negative data training sets available.

In the future, as we gain more understanding of gene regulation and additional predicted miRNA targets are experimentally validated, we expect that current limitations in miRNA target prediction tools will be addressed. For example, a method was recently proposed that takes advantage of the observation that a miRNA and its target genes are often co-regulated by common transcription factors, which may eventually be incorporated into new or current target prediction tools (Fujiwara and Yada, 2013). Currently, few of the reviewed target prediction tools are able to address tissue specificity in the prediction of miRNA targets. Tools that allow user-provided data, however, can accommodate some level of tissue specificity by incorporating tissue-specific data such as highly expressed miRNAs or miRNA isoforms, tissue-specific mRNA transcript variants, or lists of highly upregulated or downregulated genes. There is also emerging interest in integrated tools, such as miRmap (Vejnar et al., 2013), that combine multiple miRNA target identification tools to overcome the limitations of individual tools. In addition, some integrated tools, such as MiRonTop (Le Brigand et al., 2010) and CoMiR (Coronnello and Benos, 2013), incorporate expression data in the ranking of miRNA target predictions.

This review highlights the common features of miRNA target prediction and how they are incorporated into different target prediction tools. Further, we encourage the user to be aware of the version, maintenance, and data utilized for each tool. By understanding the features and the tools available, the user is well-equipped to choose the most appropriate miRNA target prediction tool available.

## Author contributions

Sarah M. Peterson, Jeffrey A. Thompson, Melanie L. Ufkin, and Clare Bates Congdon developed the concept for the structure and content of the manuscript. Sarah M. Peterson, Jeffrey A. Thompson, and Melanie L. Ufkin contributed equally to the research and initial draft of the manuscript, with guidance from Clare Bates Congdon. Sarah M. Peterson critically revised the manuscript with assistance from Jeffrey A. Thompson and input from Melanie L. Ufkin, Pradeep Sathyanarayana, Lucy Liaw, and Clare Bates Congdon. All authors reviewed and approved the final version of the manuscript.

### Conflict of interest statement

The authors declare that the research was conducted in the absence of any commercial or financial relationships that could be construed as a potential conflict of interest.
